# AI-Powered Resting 12-Lead Electrocardiogram Algorithm for Predicting Low Peak Oxygen Consumption: Development and Validation Study

**DOI:** 10.2196/81105

**Published:** 2026-06-11

**Authors:** Shu-Chun Huang, Tieh-Cheng Fu, Michelle Liou, Yu-Chieh Huang, Sing-Ya Chang, Guan-Yi Huang, Hong-Ren Su

**Affiliations:** 1 School of Medicine, College of Medicine, Taoyuan, Taiwan; 2 Department of Physical Medicine and Rehabilitation, New Taipei Municipal Tucheng Hospital, Chang Gung Memorial Hospital, Taipei, Taiwan; 3 Department of Physical Medicine and Rehabilitation, Chang Gung Memorial Hospital, Linkou, Taiwan; 4 Department of Physical Medicine and Rehabilitation, Chang Gung Memorial Hospital, Keelung City, Taiwan; 5 Institute of Statistical Science Academia Sinica, Taipei, Taiwan; 6 Department of Physical Therapy and Assistive Technology, National Yang Ming Chiao Tung University, Taipei, Taiwan; 7 Department of Medical Education, Linkou Chang Gung Memorial Hospital, Taoyuan City, Taiwan; 8 Super Genius Aitak Co, Ltd, No 5, Ln 347, Zhongzheng Rd, Xinzhuang Dist, New Taipei City, 242009, Taiwan, 886 2-206988

**Keywords:** cardiopulmonary exercise test, wavelet transform, deep learning, cardiorespiratory fitness, artificial intelligence, AI, gradient-boosting classifier

## Abstract

**Background:**

Low peak oxygen consumption (V̇O_2_) is associated with higher cardiovascular and all-cause mortality, while improvements in peak V̇O_2_ reduce this risk. Although early detection allows timely intervention, practical screening tools remain lacking. As electrocardiograms (ECGs) reflect both cardiac and age-related changes, they may offer a viable screening approach.

**Objective:**

This study aimed to detect low peak V̇O_2_ using resting 12-lead ECGs analyzed by a trained neural network.

**Methods:**

The low peak V̇O_2_ estimation model was developed using data from 965 individuals (n=540, 56% with cardiac or pulmonary disorders) mainly at Chang Gung Memorial Hospital, Linkou, and validated in an independent cohort of 242 individuals (n=194, 80% with cardiac disorders) at the Keelung branch. Resting ECGs were recorded immediately before cardiopulmonary exercise testing. Low peak V̇O_2_ was defined as a peak V̇O_2_ of <14 mL/kg/min.

**Results:**

The mean peak V̇O_2_ was 17.5 (SD 6.1) and 15.4 (SD 3.9) mL/kg/min in the training and validation datasets, with 27% (261/965) and 38% (92/242) classified as low peak V̇O_2_, respectively. Wavelet analysis improved model accuracy, underscoring its value for feature extraction. Three input models were compared: (1) individual characteristics (IC; age, sex, BMI, resting heart rate, and heart rate variability), (2) ECG alone, and (3) ECG plus IC. ECG alone outperformed IC, and combining both yielded the highest accuracy. For low peak V̇O_2_ prediction, ECG plus IC achieved mean area under the curve, precision, and recall values of 0.89, 0.72, and 0.72 in cross-validation, and 0.87, 0.67, and 0.61 in external validation.

**Conclusions:**

An artificial intelligence–driven ECG-based algorithm showed strong potential for screening low peak V̇O_2_, enabling early identification of individuals with low peak V̇O_2_ and facilitating timely clinical intervention.

## Introduction

Peak oxygen consumption (peak V̇O_2_) is a composite measure of oxygen transport chain efficiency and serves as an integrative marker of cardiorespiratory fitness. Substantial evidence has firmly established the association between low peak V̇O_2_ and high risk of cardiovascular disease and all-cause mortality [1-4]. Low peak V̇O_2_ is also linked to a decreased quality of life and a decline in functional abilities for activities of daily living. Importantly, improvement in peak V̇O_2_ is linked to lower mortality rates and enhanced quality of life [5-8]. The timely identification of high-risk patients enables the implementation of various pharmaceutical, cardiac, exercise, and nutritional interventions. Additionally, peak V̇O_2_ has been demonstrated to be a key predictor of surgical complications in thoracic surgeries and is useful for preoperative risk stratification [9,10]. Although early identification of low peak V̇O_2_ is beneficial, peak V̇O_2_ assessment requires cardiopulmonary exercise testing (CPET), which is a time-consuming and resource-intensive procedure, making it uncommon in clinical practice. Therefore, a simple and cost-effective screening tool is warranted; however, such a tool remains lacking and represents an unmet need.

It is reasonable to speculate that electrocardiogram (ECG) data could potentially serve as a screening tool for low cardiorespiratory fitness. First, previous cross-sectional and longitudinal studies have reported that age-related changes in ECG patterns include a decrease in R and S wave amplitudes, a leftward shift in frontal plane axis measurements, an increase in P-R and Q-T interval durations, a shortening of QRS duration, and a decrease in T wave amplitude [11]. Moreover, the prevalence of ECG abnormalities increases with age [12]. Second, several studies have further reported that artificial intelligence (AI) algorithms based on 12-lead ECGs can detect a reduced left ventricular ejection fraction (LVEF) [13-15]. Third, ECG abnormalities can be observed in a wide range of noncardiac conditions, including electrolyte imbalances, central nervous system diseases, and others [16]. These conditions, which are associated with ECG characteristics, simultaneously affect cardiorespiratory fitness. To the best of our knowledge, only one published study has investigated this topic [17]. However, that study used a training dataset composed primarily of relatively healthy individuals, whereas our dataset included older adults and a substantially higher proportion of patients with cardiopulmonary diseases. Further details are provided in the Discussion section.

We hypothesized that resting 12-lead ECG signals can be used to estimate individuals with low peak V̇O_2_ through a properly trained neural network model. To test this hypothesis, we analyzed the databases of simultaneously conducted resting ECGs and CPET from 2 hospitals over a 13-year period. The aim of this study was to use resting ECGs for screening individuals with low peak V̇O_2_ in a clinical setting.

## Methods

### Ethical Considerations

This research was approved by the Research Ethics Committee of the Chang Gung Medical Foundation (202301068B0C501). A waiver of consent was granted by the institutional review board. To maintain confidentiality, participants were identified only by numbers, and all research data were securely stored.

### Database

CPET data at the Chang Gung Memorial Hospital in Linkou and Keelung from 2010 to 2022 were retrieved. A 1-minute resting 12-lead ECG was performed immediately before CPET. Low peak V̇O_2_ was defined as a peak V̇O_2_ of <14 mL/kg/min, a clinical threshold indicating markedly impaired cardiorespiratory fitness and poor prognosis [11,18-20]. Data from Chang Gung Memorial Hospital, Linkou (2010‐2022; n=832) and Keelung (2010‐2013; n=133) were used as the training dataset. The data from Keelung (2014‐2022; n=242) were used for the validation dataset.

### Cardiopulmonary Exercise Testing

A symptom-limited incremental exercise test was conducted in an upright position on a calibrated cycle ergometer (Ergoselect 150P; ergoline GmbH) to evaluate cardiorespiratory fitness. The CPET was performed 2 to 4 hours after a light meal. The protocol began with a 1-minute rest period followed by a 1-minute warm-up at 10 W. The workload was then progressively increased at a rate of 10 W per minute until the participant reached exhaustion. V̇O_2_ was recorded on a breath-by-breath basis using a computerized system (MasterScreen CPX; Cardinal Health), with data averaged every 15 seconds. Resting and exercise 12-lead ECGs were monitored via a stress ECG recorder (CardioSoft; GE Healthcare), arterial blood pressure was measured using an automated device (Tango; SunTech Medical), and arterial oxygen saturation was tracked using a pulse oximeter (model 9500; Nonin Onyx). The exercise test was terminated if any of the following conditions occurred: (1) the participant was unable to maintain a pedaling frequency of 50 rpm or greater, (2) the participant reached volitional fatigue or requested to stop, (3) a plateau or decline in peak V̇O_2_ was observed despite continued effort, or (4) a cardiovascular event occurred [5,21].

### ECG Data Processing

**Figure 1.**
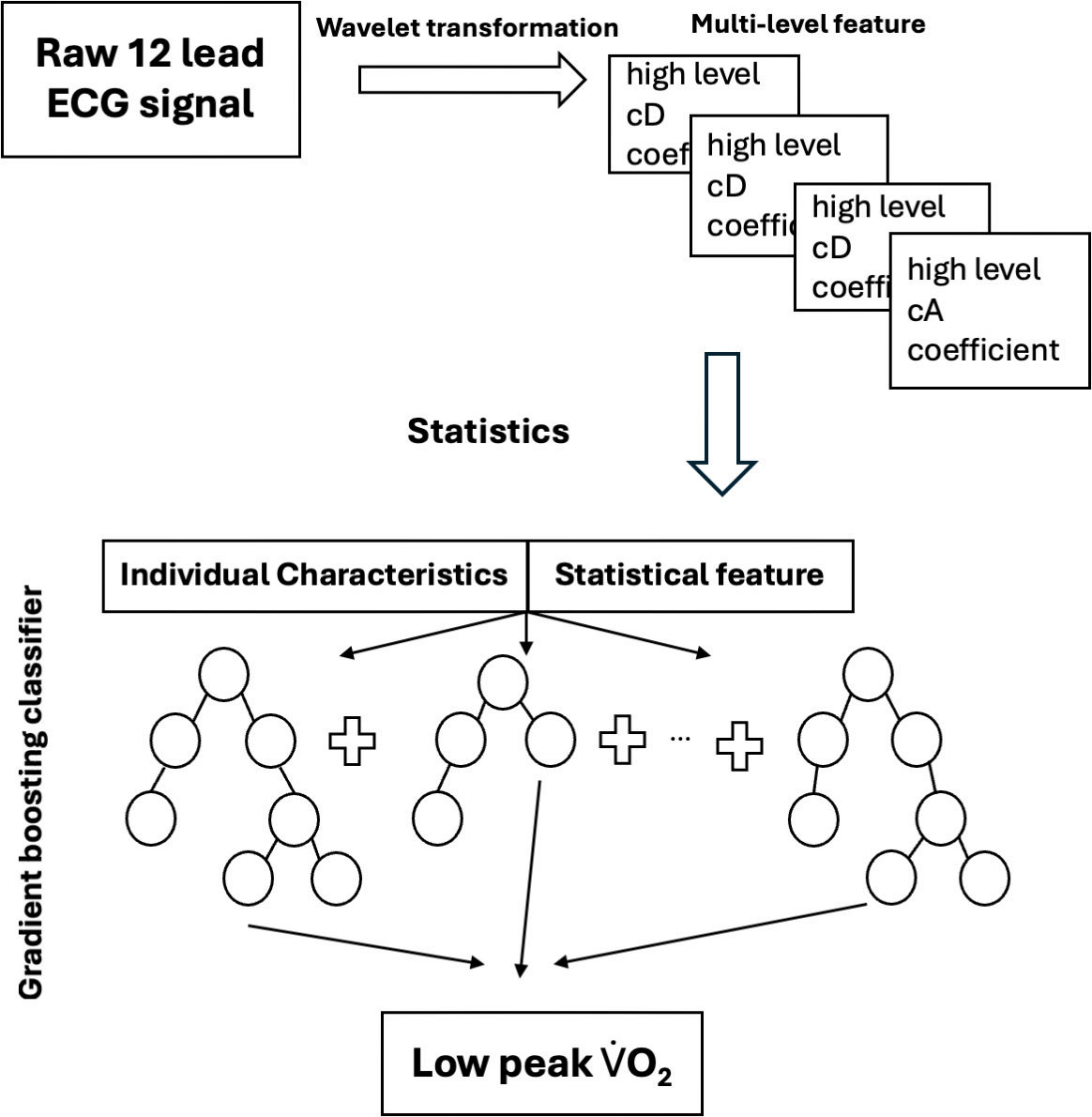
Mathematical data processing flowchart. This method analyzes 12-lead electrocardiogram (ECG) time-series data by first applying a 3-layer wavelet transform to decompose the signal into different frequency components, capturing both low-frequency rhythms and high-frequency variations. The extracted wavelet coefficients serve as features for further analysis. A gradient boosting classifier then learns from these features, using multiple decision trees to enhance prediction accuracy. This approach enables the classification of ECG signals and the prediction of low peak oxygen consumption (V̇O_2_), which is crucial for assessing cardiovascular risk and cardiorespiratory fitness. cA: high-frequency coefficient of wavelet; cD: low-frequency coefficient of wavelet.

The ECG electronic signal is a 1D time-series data sampled at a rate of 200 Hz, consisting of continuous cardiac beats that form repetitive wave groups as the analyzed data. Standard preprocessing steps for ECG signal analysis were applied. A bandpass filter (0.5‐40 Hz) was used to remove baseline drift and high-frequency noise while preserving the main frequency components of the ECG waveform. Baseline correction was then performed to eliminate low-frequency fluctuations and stabilize the signal for more reliable feature extraction [22-24]. To preserve the integrity of the original ECG data as much as possible, we applied a 3-level decomposition using the biorthogonal wavelet to process the signal [25,26] (Figure 1).

### Three Types of Training Sets

We trained the mathematical model for calculating low peak V̇O_2_ using (1) individual characteristics (IC; age, sex, BMI, resting heart rate, and heart rate variability), (2) the 60-second 12-lead resting ECG performed immediately before the CPET, and (3) IC plus 60-second 12-lead resting ECG data. Resting heart rate was defined as the average heart rate during the last 15 seconds of a 1-minute resting period. Heart rate variability was calculated from ECG signals recorded during a 1-minute resting period, based on the SD of the normal-to-normal intervals.

### Wavelet Transform for Multiresolution Analysis of Physiological Signals

Wavelet transform is an effective frequency-domain analysis method widely used for nonstationary signals, as it is capable of capturing localized and transient features within specific time intervals [27,28]. Unlike conventional Fourier-based approaches, wavelet transform simultaneously provides time-domain and frequency-domain representations, making it suitable for analyzing physiological signals such as pulse waves and ECG signals.

In practical applications, the discrete wavelet transform (DWT) is commonly used due to its ability to retain abundant signal information while significantly reducing computational time [29]. The DWT decomposes a signal x[n] into approximation cA[s,t] and detail cD[s,t] coefficients by applying scaling functions (φ) and wavelet functions (ψ):


$$cA\left[s,t\right]=\sum x\left[n\right]\phi \left(\frac{n-t2^{s}}{2^{s}}\right)$$

$$cD\left[s,t\right]=\sum x\left[n\right]\psi \left(\frac{n-t2^{s}}{2^{s}}\right)$$

At each hierarchical level, approximation coefficients are further decomposed into approximation and detail components, forming a multiresolution analysis structure analogous to a filter bank that separates signal frequency components for both coarse and fine feature analysis [30]. Compared with traditional time-frequency methods, wavelet transform provides adaptive time-frequency resolution, enabling accurate capture of rapid temporal variations while supporting noise reduction and feature compression, thereby preserving essential physiological signal morphology and improving downstream model performance [27,28]. Moreover, because many physiological signals are inherently nonstationary, the wavelet transform is particularly well suited to capturing dynamic and transient characteristics in signals such as ECG signals [31].

In this study, biorthogonal wavelets were selected for their favorable mathematical and signal-processing properties [32]. Their symmetry and linear-phase response help minimize edge distortion and preserve waveform morphology—critical for physiological signals. They also satisfy perfect reconstruction conditions, allowing signals to be decomposed and rebuilt without distortion [33]. In addition, compared with other wavelet families such as Daubechies, biorthogonal wavelets provide more flexibility in controlling smoothness and decomposition depth, enabling more stable multiscale feature extraction.

During multiresolution decomposition, each level generates a unique set of DWT coefficients representing the signal’s characteristics at corresponding scales. To extract meaningful information, statistical measures (eg, mean, variance, energy, and entropy) are computed from these coefficients, providing a quantitative representation of the signal distribution and dynamics across resolutions [33]. These features support comprehensive and robust physiological signal characterization.

### Gradient Boosting Classifier for Physiological Signal Classification

The gradient boosting classifier (GBC) is a stage-wise ensemble learning framework that constructs a strong predictive model by sequentially combining multiple weak learners, typically small regression or classification trees [34]. Formally, the predictive function can be expressed as:


$$F\left(x;a_{n},b_{n}\right)=\sum_{n=1}^{N}b_{n}{h(x;a}_{n})$$

where *h* denotes a base learner and *b*_*n*_ is the weight assigned at each boosting stage. At each iteration, the model adds a new regression tree fitted to the negative gradient of the loss function, progressively correcting residual errors from previous stages. This iterative gradient-guided refinement enables the ensemble to capture complex patterns, nonlinear relationships, and subtle decision boundaries within physiological signal datasets [35].

GBC is well suited for physiological signal classification because it effectively captures nonlinear feature interactions and handles heterogeneous data. Compared with single decision trees, it offers improved accuracy and generalization through reduced bias and variance, with tunable hyperparameters and feature importance supporting model control and interpretability.

GBC can be computationally heavy, sensitive to hyperparameters and noise, and hard to interpret, yet it remains effective for classifying variable or noisy physiological signals in biomedical applications. In this study, the key hyperparameters of the GBC, such as tree depth, learning rate, and number of iterations, were optimized using systematic tuning combined with cross-validation. The model used shallow trees, a small learning rate, and cross-validated iterations to balance nonlinear modeling and prevent overfitting. Improper hyperparameters can cause overfitting or underfitting, so careful tuning is essential for stable performance. On the basis of this strategy, we believe that the selected hyperparameter combination enables the GBC to classify ECG signals effectively while maintaining stability and generalizability.

### Resampling Strategy

The prevalence of low peak V̇O_2_ in this dataset is 29%. Training a model on such imbalanced data may introduce bias toward the majority class, potentially overlooking important information within the minority class and compromising the reliability of model predictions. To address this issue, we used a data resampling strategy. Specifically, we used oversampling of the minority class to balance the number of samples across different classes. The resampling ratio was determined according to the original class distribution (27%) in the training dataset, aiming to improve the model’s ability to recognize the minority class while preserving the integrity of the original data. Oversampling substantially improved the model’s classification performance for the minority class, leading to enhanced overall accuracy and greater performance stability. This methodological choice is supported by prior studies, which also suggest that oversampling is a simple yet effective strategy for addressing class imbalance in small-sample physiological signal classification tasks [36,37].

In our resampling approach, each class was represented by a comparable number of data points during training, balancing the influence of each class and allowing the model to learn effectively from both majority and minority classes. To ensure robustness, multiple experiments were conducted by repeating the process and meticulously recording statistical results. This iterative procedure demonstrates the consistency and reliability of our method across trials, highlighting its effectiveness in handling imbalanced datasets.

Additionally, the wavelet transform was compared with the outcomes obtained using a 1D convolutional neural network (1D CNN), based on prior research demonstrating its effectiveness in extracting key features from ECG data and achieving high classification accuracy [38]. This 1D CNN used a standard architecture and parameter settings that have been widely validated in ECG analysis. It comprised 2 convolutional layers, 2 downsampling layers, and a fully connected layer, with ECG data processed without applying a wavelet transform, allowing the model to automatically learn relevant features.

### Cross-Validation and External Validation

In the training dataset, a 4-fold cross-validation [39] was also applied to avoid overfitting and verify accuracy. It was performed by assessing model performance and comparing the numbers of true and AI-generated low peak V̇O_2_ and non–low peak V̇O_2_ cases. The original ECG data were randomly partitioned into 4 equal-sized subdatasets. Of the 4 subdatasets, a single subdataset was preserved as validation data to test the model, and the remaining 3 subdatasets were used as training data. The cross-validation process was repeated 4 times, with each of the 4 subdatasets used once as the validation data. The results were then averaged. To evaluate the model’s ability to identify individuals with markedly impaired cardiorespiratory fitness, defined as a peak V̇O_2_ of <14 mL/kg/min—a clinical threshold for substantial impairment—external validation was also conducted [19,20].

### Statistics

The validation of our model was assessed using precision, recall, and total accuracy. Precision, defined as the ratio of correctly predicted positive observations to the total number of predicted positives, measures the accuracy of positive predictions, while recall represents the ratio of correctly predicted positive observations to all observations in the actual class. Total accuracy reflects the proportion of correctly predicted observations, including both low peak V̇O_2_ and non–low peak V̇O_2_, among all observations. Additionally, a receiver operating characteristic curve analysis was conducted to determine the area under the curve (AUC) for predicting low peak V̇O_2_. All data are reported as means (SD), with statistical significance set at *P*<.05.

## Results

The training dataset comprised 965 individuals, of whom 77% (n=743) were male. The mean age and BMI were 57 (SD 13) years and 24.4 (SD 4.6) kg/m^2^, respectively. The average peak V̇O_2_ was 17.5 (SD 6.1) mL/kg/min. A total of 263 (27.2%) individuals had low peak V̇O_2_. Common morbidities potentially associated with reduced peak V̇O_2_ included heart failure (n=241, 25%), coronary artery disease (n=166, 17%), chronic obstructive pulmonary disease (n=57, 6%), esophageal cancer (n=237, 25%), and stroke (n=105, 11%; Table 1). The validation dataset included 242 individuals, with 174 (71.9%) being male participants. The mean age and BMI were 59 (SD 12) years and 25.8 (SD 4.4) kg/m^2^, respectively. The mean peak V̇O_2_ was 15.4 (SD 3.9) mL/kg/min. Among these, 91 (37.6%) cases were classified as having low peak V̇O_2_. Cardiac disorders accounted for 80% (n=194) of the patients in the dataset (Table 1).

**Figure 2.**
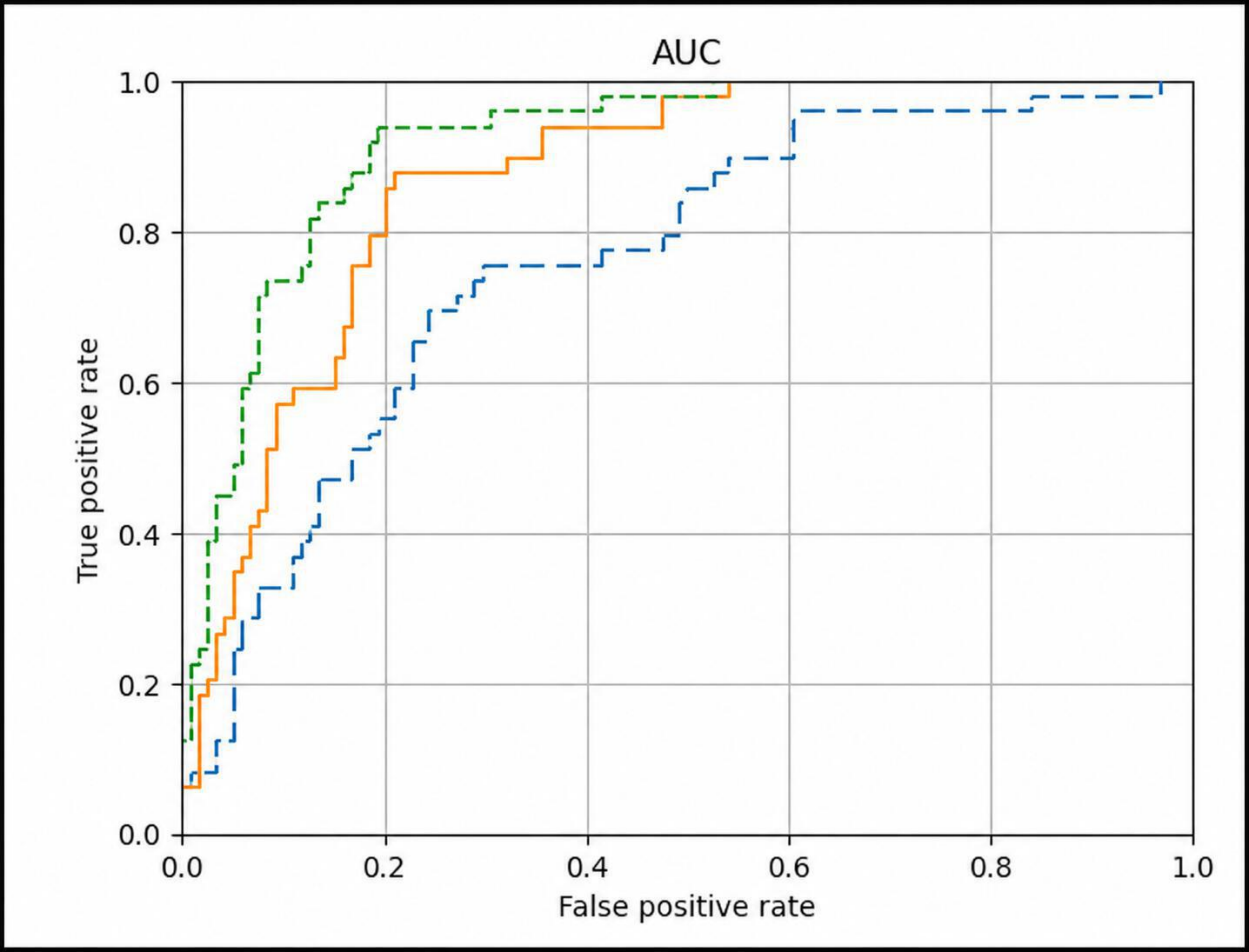
Receiver operating characteristic curves from 3 different input models as follows: individual characteristics (long-dashed blue line), electrocardiogram (ECG) alone (orange line), and individual characteristics plus ECG (short-dashed green line). All 3 curves display the best results from the 4 cross-validation tests. AUC: area under the curve.

In Table 2, the accuracy of all the parameters, when compared with the null hypothesis of random guessing, has a *P* value of <.01. Moreover, the accuracy of the AI model was higher when using wavelet analysis than when not using wavelet analysis, suggesting that wavelet analysis assisted in feature detection. ECG alone demonstrated clearly higher accuracy than IC. Incorporating IC into ECG signals led to a modest improvement in estimation accuracy in both cross-validation (Figure 2) and external validation. In the external validation, the AUC increased from 0.84 with ECG alone to 0.87 when combined with IC (Table 3).

For low peak V̇O_2_ estimation, the combination of ECGs and IC achieved a mean AUC, precision, and recall of 0.89, 0.72, and 0.72 during cross-validation, and 0.87, 0.67, and 0.61 during external validation. In other words, during external validation, the model successfully identified 61% of low peak V̇O_2_ cases (Table 3).

**Table 1.** Database attributes.

	Training set (n=965)	Validation set (n=242)
Age (years), mean (SD)	57 (13)	59 (12)
Sex		
Female participants, n (%)	222 (23)	70 (28.9)
Male participants, n (%)	743 (77)	172 (71.0)
Body height (cm), mean (SD)	165.7 (8.6)	163.2 (9.2)
Body weight (kg), mean (SD)	67.2 (15.3)	68.8 (13.3)
BMI (kg/m^2^), mean (SD)	24.4 (4.6)	25.8 (4.4)
Clinical diagnoses		
Cardiac, n (%)		
Heart failure	245 (25.4)	165 (68.2)
Coronary artery disease	166 (17.2)	118 (48.8)
ST-elevation myocardial infarction	42 (4.4)	45 (18.6)
Non–ST-segment elevation myocardial infarction	14 (1.5)	11 (4.5)
Post–coronary artery bypass grafting	37 (3.8)	48 (19.8)
Valvular heart disease	62 (6.4)	30 (12.4)
Dilated cardiomyopathy	50 (5.2)	5 (2.1)
Peripheral artery disease	6 (0.6)	2 (0.8)
Myocarditis	8 (0.8)	0 (0)
Endocarditis	6 (0.6)	0 (0)
Pulmonary, n (%)		
Chronic obstructive pulmonary disease	57 (5.9)	23 (9.5)
Pneumoconiosis	85 (8.8)	0 (0)
Lung cancer	63 (6.5)	0 (0)
Interstitial lung disease	29 (3)	0 (0)
Idiopathic pulmonary hypertension	8 (0.8)	0 (0)
Pneumothorax s/p	8 (0.8)	0 (0)
Obstructive sleep apnea	12 (1.2)	16 (6.6)
Others, n (%)		
Esophageal cancer	237 (24.6)	0 (0)
Head and neck cancer	24 (2.5)	0 (0)
Hypertension	326 (33.8)	141 (58.3)
Diabetes mellitus	194 (20.1)	84 (34.7)
Hyperlipidemia	134 (13.9)	101 (41.7)
Chronic kidney disease	90 (9.3)	27 (11.2)
Gout	61 (6.3)	20 (8.3)
Stroke	105 (10.9)	23 (9.5)
Alcoholism	127 (13.2)	0 (0)
Liver cirrhosis	119 (12.3)	21 (8.7)
Systemic lupus erythematosus	3 (0.3)	0 (0)
Healthy participants, n (%)	38 (3.9)	0 (0)
True peak V̇O_2_^a^ (mL/kg/min), mean (SD)	17.5 (6.1)	15.4 (3.9)
Number of low peak V̇O_2_, n (%)	263 (27.3)	91 (37.6)

a V̇O_2_: oxygen consumption.

**Table 2.** Cross-validation accuracy of the training dataset (n=965) across 3 input types.^a^

	With wavelet analysis						Without wavelet analysis	
	IC^b^, mean (SD)		ECG^c^ alone, mean (SD)		ECG plus IC, mean (SD)		ECG plus IC, mean (SD)	
Precision of low peak V̇O_2_^d^	0.61 (0.05)		0.70 (0.06)		0.72 (0.05)		0.34 (0.07)	
Recall of low peak V̇O_2_	0.61 (0.04)		0.72 (0.05)		0.72 (0.06)		0.92 (0.05)	
Total accuracy	0.81 (0.02)		0.83 (0.03)		0.83 (0.02)		0.61 (0.03)	
AUC^e^	0.77 (0.03)		0.89 (0.02)		0.89 (0.02)		0.84 (0.03)	
Precision of non–low peak V̇O_2_	0.84 (0.02)		0.88 (0.02)		0.89 (0.03)		0.96 (0.01)	
Recall of non–low peak V̇O_2_	0.84 (0.04)		0.88 (0.02)		0.89 (0.03)		0.55 (0.03)	

a When computing the area under the curve, “positive” refers to low peak oxygen consumption.

b IC: individual characteristics.

c ECG: electrocardiogram.

d V̇O_2_: oxygen consumption.

e AUC: area under the curve.

**Table 3.** Model performance on external validation (n=242).

	With wavelet analysis			Without wavelet analysis
	IC^a^	ECG^b^ alone	ECG plus IC	ECG plus IC
Precision of low peak V̇O_2_^c^	0.51	0.64	0.67	0.27
Recall of low peak V̇O_2_	0.47	0.63	0.61	0.83
Total accuracy	0.77	0.79	0.82	0.54
AUC^d^	0.73	0.84	0.87	0.79
Precision of non–low peak V̇O_2_	0.81	0.84	0.85	0.92
Recall of non–low peak V̇O_2_	0.79	0.83	0.85	0.45

a IC: individual characteristics.

b ECG: electrocardiogram.

c V̇O_2_*:* oxygen consumption.

d AUC: area under the curve.

## Discussion

### Principal Findings

This study demonstrated that a complete resting ECG signal can effectively screen individuals with low cardiorespiratory fitness using a well-trained neural network. Wavelet-based feature extraction contributed to greater accuracy in AI models. For low peak V̇O_2_ estimation, combining ECGs with IC, including age, sex, BMI, resting heart rate, and heart rate variability, yielded an AUC of 0.87, which is considered acceptable for clinical use. The AI model used wavelet transform for feature extraction, a GBC for prediction, and resampling techniques to address imbalanced data. Notably, ECG alone outperformed IC in estimation accuracy, while the addition of IC to ECGs resulted in a modest improvement. Overall, resting ECGs, either alone or combined with IC, serve as a valuable tool for identifying individuals with low cardiorespiratory fitness with satisfactory accuracy. Early identification of this at-risk population enables timely medical intervention and may improve prognosis.

To the best of our knowledge, our research is the second study that has investigated the use of ECGs to calculate peak V̇O_2_. The first study demonstrated the feasibility of estimating peak V̇O_2_ from ECGs using deep learning techniques trained on 1410 samples and validated on 3 different sets [28]. The present experimental findings largely reproduce the results of this previous study, confirming that resting ECG-based deep learning is a noteworthy new approach to screen for low peak V̇O_2_. The difference from previous research lies in the composition of the current database, which mostly includes patients with frailty and ailments. The peak V̇O_2_ in this dataset was 17.5 mL/kg/min, compared with 33.6 mL/kg/min in the Massachusetts General Hospital (MGH) training set [28]. We observed a higher prevalence of peak V̇O_2_ at <14 mL/kg/min in this study than in the MGH training set (27% vs 8.5%). The mean age of the patients in this dataset was 57 (SD 13) years, whereas that in the MGH training dataset was 45 years. In addition, differences in the distribution of clinical traits were observed between the patient populations in the 2 studies. For instance, the present database includes a higher proportion of patients with cardiac or pulmonary diseases (Table 1). Specifically, the prevalence of heart failure was 25% (245/965) in the training dataset and 68% (165/242) in the validation dataset, compared with only 4% in the MGH training dataset. It is also worth mentioning that this method still cannot accurately predict the absolute value of peak V̇O_2_. The true estimation error of peak V̇O_2_ by the ECG-deep learning method is quite wide. The lower and upper limits of agreement range from −14.95 to 15.08 and from −17.6 to 10.5 mL/kg/min in the 2 test sets [28]. This study also reported that individuals with higher peak V̇O_2_ seem to exhibit greater R and S wave amplitudes, as well as more pronounced ST segments and T waves [28]. In conclusion, our study further illustrates that resting ECGs by deep learning could still identify individuals with low peak V̇O_2_ in a vulnerable population. Accordingly, this approach may influence clinical decision making and warrants further investigation. The value of this method is particularly highlighted in patients who have conditions that preclude them from undergoing CPET, such as musculoskeletal disorders, frailty, and others.

Peak V̇O_2_ represents the integrative performance of the oxygen delivery and use system. Cardiac electrical signals may, to some extent, reflect certain aspects of this system. Therefore, from a physiological standpoint, resting ECGs may not precisely predict the exact value of peak V̇O_2_, although it remains effective in identifying individuals with low peak V̇O_2_.

Current studies demonstrate that incorporating IC leads to only modest enhancements in estimation accuracy. This can be explained by a previous study illustrating that applying AI to ECG data enables the prediction of sex and age [29]. In addition, ECGs provide various biological data. Several studies have reported that using an AI algorithm based on a 12-lead ECG can enable the early diagnosis of low LVEF defined by an LVEF of ≤35% in patients in the routine primary care setting [13-15]. ECGs also predict the onset of various cardiovascular diseases, including atrial fibrillation, myocardial infarction, heart failure, and death [15,28]. Moreover, ECGs show promise in detecting dysglycemia [28,31,32]. Furthermore, blood lactate concentration during exercise was accurately estimated from exercise ECG data using 2D convolution and artificial neural network–based methods [26]. Collectively, these studies underscore the wealth of biological information in ECG data that awaits further exploration.

A standard resting ECG is a simple and widely used clinical examination that is routinely performed in patients with cardiovascular disease or as part of routine preoperative assessment. The ECG-based algorithm developed in this study shows strong potential as a large-scale screening tool for identifying individuals at high risk of low peak V̇O₂. For clinical implementation, the algorithm could be directly integrated into the GE ECG autointerpretation system or embedded within ECG recorders from partnering manufacturers. In the latter case, appropriate calibration—such as matching the sampling frequency—would be required to maintain accuracy and reliability, followed by further validation.

On the basis of this result, when the raw ECG signals were directly input into the 1D CNN, the classification performance decreased compared with using features extracted via wavelet transform. The main reasons are as follows: first, raw ECG signals exhibit high-dimensional and nonlinear characteristics, with transient waveform variations and multifrequency components, which can make it difficult for a 1D CNN to effectively capture all relevant features when the signals are used directly; and second, the wavelet transform provides joint time-frequency information by converting raw ECG signals into informative multiscale features that emphasize key transient waveforms and frequency components, which are more easily exploited by downstream classifiers (such as GBC or 1D CNN), thereby improving classification performance. In addition, previous studies have shown that time-frequency analysis methods, such as the wavelet transform, can significantly improve the classification accuracy of machine learning and deep learning models applied to ECG signals [22,40].

In summary, this study demonstrates that resting ECGs, either alone or combined with IC, can effectively identify individuals with low cardiorespiratory fitness using a deep learning framework. Wavelet-based feature extraction contributed to the model’s accuracy, and the GBC provided robust prediction capabilities for low peak V̇O_2_ estimation. While ECGs combined with IC yielded an AUC of 0.87, ECG alone already performed strongly, indicating its potential as a practical screening tool. Compared with other classifiers such as support vector machines or random forests, GBC offers several advantages for ECG analysis: it can automatically model high-dimensional, nonlinear, and transient features; it demonstrates stability and generalization for heterogeneous, small, or noisy datasets; and it provides feature importance insights that help interpret which waveform patterns are most influential [41]. Prior studies have also reported that GBC or its variants (eg, Extreme Gradient Boosting) achieve high accuracy in ECG classification or arrhythmia detection, often outperforming support vector machines and random forests [42]. Accordingly, the combination of wavelet transform for feature extraction and GBC for classification represents a methodologically sound and effective approach.

Our findings further reproduce and extend previous studies on ECG-based estimation of peak V̇O_2_, highlighting its utility even in populations with a high burden of comorbidities. Although resting ECGs cannot precisely predict absolute peak V̇O_2_ values, it effectively identifies at-risk individuals who may benefit from timely intervention. This approach is particularly valuable for patients who cannot undergo CPET due to musculoskeletal disorders, frailty, or other limitations. Future work may focus on further refining model accuracy, expanding validation in diverse populations, and exploring integration into clinical ECG systems for real-world screening and decision support.

Regarding the slight decrease in accuracy observed in the external validation set, several factors may account for this phenomenon. First, the external validation data were collected using different devices and obtained from distinct subject populations, which may result in subtle differences in signal characteristics compared with the training dataset. Such cross-dataset performance fluctuations are commonly reported in ECG-based machine learning studies and do not necessarily indicate model instability. Second, the GBC model is inherently sensitive to high-dimensional and nonlinear features derived from multiscale wavelet transforms. Although these features substantially enhance overall classification performance, they may also amplify the impact of minor distributional differences between datasets. As a result, even small discrepancies in signal morphology or noise characteristics can lead to a modest reduction in accuracy when the model is applied to an external cohort. Overall, the slight decrease in accuracy in the external validation set is a common issue in physiological signal analysis and does not compromise the methodological validity or the main conclusions of our ECG pattern recognition model.

### Limitation

First, incorporating other factors such as the severity of comorbidities could potentially enhance the model’s accuracy. However, this study focuses on clinical application, where usability is just as crucial as accuracy. Therefore, we prioritize easily accessible input parameters, such as the IC defined in this study. With the current model, we achieve both acceptable accuracy and practical usability, with an AUC of 0.84 using ECG alone and 0.87 when combining ECGs with IC. Second, the dataset comprised mostly of East Asian individuals with a high prevalence of morbidities; therefore, caution is needed when applying the algorithm to other populations. Third, we did not specifically analyze the relationships between individual ECG features and low peak V̇O_2_, because the model is designed to automatically extract multiscale, high-dimensional features that capture global ECG waveform patterns, rather than one-to-one associations between single features and clinical traits.

### Conclusions

This study demonstrated that a 12-lead resting ECG, a widely accessible test, can effectively identify individuals with a peak V̇O_2_ of less than 14 mL/kg/min when used in a well-trained neural network, with satisfactory accuracy. Wavelet feature extraction significantly enhanced the performance of AI models. This approach has the potential to improve patient care by facilitating earlier identification and intervention for individuals with markedly reduced cardiorespiratory fitness and an elevated risk of cardiovascular events.

## Abbreviations

1D CNN: 1D convolutional neural network
AUC: area under the curve
CPET: cardiopulmonary exercise testing
DWT: discrete wavelet transform
ECG: electrocardiogram
GBC: gradient boosting classifier
IC: individual characteristics
LVEF: left ventricular ejection fraction
MGH: Massachusetts General Hospital
V̇O_2_: oxygen consumption
